# Recent studies on berberine and its biological and pharmacological activities

**DOI:** 10.17179/excli2022-5898

**Published:** 2023-02-28

**Authors:** Priscilla Nadalin, Yong-Goo Kim, Sang Un Park

**Affiliations:** 1 Department of Crop Science, Chungnam National University, 99 Daehak-ro, Yuseong-gu, Daejeon, 34134, Korea; 2 Department of Herbal Crop Research, National Institute of Horticultural and Herbal Science, RDA, Eumseong 27709, Korea

## ⁯

Benzylisoquinoline alkaloids (BIAs) are a variety of plant chemicals that consist of roughly 2,500 known compounds. Many BIAs are characterized by potent pharmacological activities, notably the narcotic analgesics morphine and codeine, the antimicrobials sanguinarine and berberine, the muscle relaxants (+)-tubocurarine and papaverine, and the cough suppressant and anticancer drug noscapine. A number of medicines that have been known to humankind since ancient times are plant-derived BIAs (Hagel and Facchini, 2013[26]).

Berberine (BBR), a yellowish crystalline benzylisoquinoline alkaloid, is an active compound found in several plants. BBR has been used in traditional Chinese medicine for a long time to treat several conditions (Zhu et al., 2022[107]). BBR alkaloids are found in the leaves, bark, twigs, rhizomes, roots, and stems of plants, with bark and roots containing reasonably high amounts of BBR in comparison to other plant parts (Andola et al., 2010[7]). BBR is a tetracyclic ring system consisting of an N-benzyltetrahydroisoquinoline core with an incorporated additional C13 carbon bridge, which is formed through an oxidative step where the N-methyl group is provided by S-adenosyl methionine to an iminium ion, with a consequent cyclization into an aromatic ring through the phenolic hydroxyl. Starting from tyrosine, BBR biosynthesis consists of 13 stages in which various enzymatic reactions are involved (Singh et al., 2021[70]). 

BBR has been reported to be useful for a wide range of biological and pharmacological activities, including antioxidant, anti-inflammatory, anticancer, antimicrobial, antidepressant, hepatoprotective, hypolipidemic, and hypoglycemic activities (Almatroodi et al., 2022[5]; Behl et al., 2022[8]; Cheng et al., 2022[11]; Och et al., 2022[54]; Yarmohammadi et al., 2022[93]; Mohammadian Haftcheshmeh and Momtazi‐Borojeni, 2021[48]). Interestingly, many studies have provided evidence suggesting that BBR is a valuable drug candidate with a wide range of therapeutic uses (Mujtaba et al., 2022[49]). Here, we report a summary of the current literature available on the biological and pharmacological activities of BBR (Table 1(Tab. 1); References in Table 1: Abdulredha et al., 2021[1]; Abudureyimu et al., 2020[2]; Akhzari et al., 2022[3]; Albasher et al., 2020[4]; An et al., 2022[6]; Chen et al., 2021[10], 2022[9]; Cole et al., 2021[12]; Cui et al., 2022[13]; Dai et al., 2021[14], 2022[15]; Deng and Ma, 2021[16]; Deng et al., 2022[17]; Diao et al., 2022[18]; Ding et al., 2021[19]; Duarte-Olivenza et al., 2021[20]; Ehteshamfar et al., 2020[21]; Fang et al., 2022[22]; Fu et al., 2020[23]; Ghanem et al., 2021[24]; Gu et al., 2021[25]; Han et al., 2022[27]; He et al., 2021[28]; Huang et al., 2021[30], 2022[29]; Ibrahim Fouad and Ahmed, 2021[31]; Jia et al., 2022[32]; Jiang et al., 2021[33]; Kadir et al., 2022[34]; Kang et al., 2020[35]; Li et al., 2020[38], 2021[36], 2022[37]; Liang et al., 2021[39]; Liu et al., 2022[40]; Luo et al., 2022[41]; Lv et al., 2022[42]; Ma et al., 2021[43], 2022[44]; Malekinezhad et al., 2021[45]; Man et al., 2022[46]; Mishra et al., 2020[47]; Ni et al., 2022[50][51]; Noh et al., 2022[52]; Obchoei et al., 2022[53]; Pan et al., 2022[55]; Pang et al., 2021[56]; Park et al., 2022[57]; Pu et al., 2021[58]; Qiu et al., 2021[59]; Qu et al., 2020[60]; Ren et al., 2021[61]; Rong et al., 2022[62]; Seth and Chopra, 2022[64]; Seth et al., 2021[63]; Shaker et al., 2021[65]; Shan et al., 2021[66]; Shao et al., 2021[67]; Shen et al., 2021[68]; Shu et al., 2021[69]; Sui et al., 2021[71]; Sun et al., 2022[72]; Szalak et al., 2021[73]; Tang et al., 2021[74]; Tian et al., 2021[75], 2022[76]; Vita et al., 2021[77]; Waly et al., 2022[78]; Wang et al., 2021[80], 2022[79]; Wen et al., 2022[81]; Wolf et al., 2021[82]; Wu et al., 2021[83]; Xia et al., 2022[84]; Xiang et al., 2021[85]; Xie et al., 2020[86], 2021[88], 2022[87]; Xu et al., 2021[89]; Yan et al., 2022[90]; Yang et al., 2021[92], 2022[91]; Ye et al., 2021[94], 2022[95]; Yi et al., 2021[96]; Yin et al., 2021[97]; Yu et al., 2021[98]; Zhai et al., 2020[99][100]; Zhang et al., 2022[101][102]; Zhao et al., 2021[103]; Zheng et al., 2021[104][105]; Zhu et al., 2021[106]).

## Notes

Priscilla Nadalin and Yong-Goo Kim contributed equally as first author.

## Declaration

### Conflict of interest statement

The authors have no conflicts of interest to declare.

### Acknowledgments

This research was supported by Basic Science Research Program through the National Research Foundation of Korea (NRF) funded by the Ministry of Education (2022R1I1A3054240)

## Figures and Tables

**Table 1:**
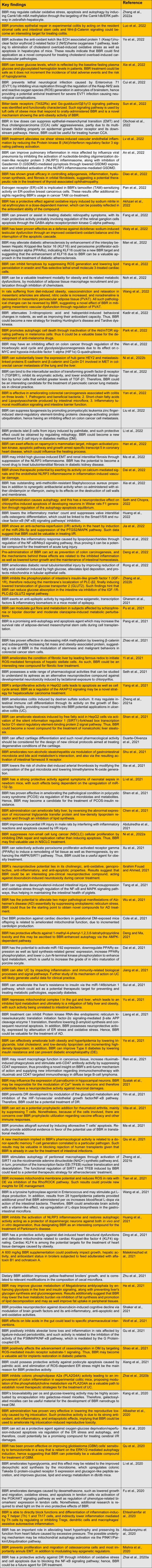
Recent studies on the biological and pharmacological activities of berberine
